# IgE Sensitization to the Nonspecific Lipid-Transfer Protein Ara h 9 and Peanut-Associated Bronchospasm

**DOI:** 10.1155/2013/746507

**Published:** 2013-09-12

**Authors:** Peter D. Arkwright, Colin W. Summers, Beverley J. Riley, Najla Alsediq, Richard S. H. Pumphrey

**Affiliations:** ^1^Institute of Inflammation and Repair, University of Manchester, Manchester M13 9WL, UK; ^2^Department of Paediatric Allergy & Immunology, Royal Manchester Children's Hospital, University of Manchester, Oxford Road, Manchester M13 9WL, UK; ^3^Department of Immunology, Salford Royal Foundation Trust, Manchester M6 8HD, UK

## Abstract

Allergen component analysis is now available in many laboratories. The aim of this study was to examine the possible association between peanut allergen IgE components and severity of clinical reactions in patients with a history of peanut allergy. Data and sera collected from 192 patients within the Manchester Allergy Research Database and Serum Bank were used in this retrospective study. Sensitization to peanut specific IgE and Ara h 1, 2, 3, and 8 peanut IgE components, as measured by fluoroenzyme immunoassay, was not associated with anaphylaxis. In contrast, sensitization to the lipid-transfer protein Ara h 9 was significantly more prevalent in patients with peanut-associated bronchospasm (26% versus 9% of patients), even after adjusting for potential confounding effects of age, gender, and severity of concomitant chronic atopic diseases. Patients who were sensitized to Ara h 9 were more likely to have ingested rather than just have had skin contact with peanut and have a more rapid onset of symptoms. These results are consistent with observations that sensitization to heat and protease resistant lipid-transfer protein components of hazelnut, grains, and fruit is predictive of anaphylaxis.

## 1. Introduction

Approximately 1/100 individuals in Western countries suffer from peanut allergy [1]. Peanut is a leading cause of food-induced anaphylaxis, defined as a serious allergic reaction that is rapid in onset and may cause death through respiratory or circulatory failure [2–4]. Adolescences and young adults, as well as those with severe asthma, are prone to more severe reactions [5]. Skin prick tests and serum specific IgE concentrations are markers of immune sensitization. Thirteen specific allergic protein components of peanut have now been identified and characterized [6, 7]. Ara h 2 is the predominant antigen in peanut-allergic patients in some but not all countries. It may also help to predict the likelihood of clinical reactivity in patients without a clinical history of peanut allergy [8–11]. Ara h 1, 3, and 6 are other seed storage proteins that commonly induce IgE responses [12, 13]. Ara h 8 is a heat and enzyme-labile pathogenesis-related protein (PR-10) with cross-reacting antigenic epitopes to birch tree pollen allergen Bet v 1. Because of its degradation by salivary and gastric juices, IgE-mediated reactions to PR-10 allergens are commonly localized to the oral cavity [14–16]. In contrast, Ara h 9 is a heat and enzyme-stable nonspecific lipid transfer protein (nsLTP) with cross-reacting epitopes to other nsLTP such as hazelnut (Cor a 8) and peach (Pru p 3) [17].

Allergen analysis to these components is increasingly available in clinical immunology laboratories around the world. There are data to suggest that this technology might predict peanut allergy in selected patients without the need for oral challenges [8, 18]. However, at present there is no evidence that it can predict the risk of life-threatening anaphylaxis. The Manchester Allergy Research Database has detailed, standardised, clinical data on the severity of clinical reactions in patients with peanut allergy. The current study aims to address the question as to whether component analysis can predict the risk of anaphylaxis defined as clinical features of respiratory or circulatory compromise in patients with documented clinical peanut allergy. 

## 2. Experimental

### 2.1. Patients

Clinical details of patients with peanut allergy attending a regional allergy clinic at Manchester Royal Infirmary, Manchester, UK between 1992 to 2004 were recorded onto structured history forms by allergy specialists (PDA and RSP). All patients were referred by primary care physicians or general pediatricians. The diagnosis was made on the basis specialists' clinical history of allergic reaction to peanuts or peanut containing food taken at the consultation, supported by either evidence from skin prick tests or raised allergen specific serum IgE concentrations. Allergic symptoms were carefully documented, particularly acute respiratory or circulatory symptoms occurring after eating the food indicative of more serious reactions (anaphylaxis). Pharyngeal edema was considered in patients with a hoarse voice, difficulty swallowing, and/or difficulty breathing. Bronchospasm was considered if patients were wheezy. Reduced (dizziness/light headedness) loss of consciousness were considered features of circulatory insufficiency. 

Patients were classified as having atopic dermatitis if they fulfilled the British Association of Dermatologists Working Party Criteria and required topical corticosteroids or calcineurin antagonists for control of their symptoms. Patients with moderate-severe asthma were those with wheeze requiring inhaled corticosteroids and/or leukotriene antagonists in addition to inhaled bronchodilators. Patients with allergic rhinitis had sneezing, itchy eyes, nasal congestion, and rhinorrhea requiring oral antihistamines or topical intranasal steroids. Clinical details were also recorded as to the date of reaction in relation to consultation, the amount of food consumed and the delay between consuming the food and the onset of the reaction. 

All patients were otherwise well and were not on regular *β*-blockers, other antihypertensives, or antidepressants. Serum for measurement of total and allergen specific IgE (sIgE) was taken at the initial consultation and stored at −40°C in a centralized serum bank before processing. All patients signed a written consent form, and the collection of data and serum had the approval of the Local Research Ethics Committee (04/Q1401/44). 

### 2.2. Serum IgE Measurements

Total serum IgE concentrations, peanut (peanut specific, Ara h 1, Ara h 2, Ara h 3, Ara h 8, Ara h 9 allergen components), tree nut specific (almond, brazil, cashew, hazelnut, cashew), and cross-reacting allergen component (Bet v 1 from birch, Cor a 1 and Cor a 8 from hazelnut, Pru p 3 from peach) IgE were measured by fluoroenzymeimmunoassay using the automated ImmunoCAP250 processor (Thermo Scientific (formerly Phadia Ltd), UK). Timothy grass pollen components Phl p 1, 4, 5B, 7, and 12 were also measured. Total IgE is quoted as kilounits per litre (kU/L) and sIgE units as allergen specific kilounits per litre (kU_A_/L). Sensitization was defined as patients with peanut or peanut component sIgE concentrations ≥0.35 kU_A_/L.

### 2.3. Statistical Analysis

Data from the centralized Access database were entered into an SPSS spreadsheet (IBM SPSS Statistics 20, Chicago, Ill). The sample size had an 80% power to detect 2.5-fold differences between patients with and without symptoms of anaphylaxis with a two-tailed *P* value of 0.05. As some data were not normally distributed, continuous variables are quoted as median (interquartile range). Initially, Chi-square and Mann-Whitney *U* tests were used to detect statistical differences between groups. Covariant analyses examining the relative effect of a number of variables at the same time as shown in Tables 2 and 3 were performed using multinominal logistic regression. In this statistical model, symptoms of (i) pharyngeal edema (none, hoarse voice (mild), or drooling and dyspnea (moderate-severe)); (ii) bronchospasm (none, wheeze (mild), wheeze with dyspnea (moderate-severe)); or (iii) circulatory insufficiency (none, dizziness (mild), loss of consciousness (moderate-severe)) were the discrete independent variable used in three separate analyses. Discrete covariants used in the regression equations were: child or adult; gender; atopic dermatitis; asthma; allergic rhinitis; sensitization (defined as ≥0.35 kU_A_/L) to peanut IgE, peanut allergen components Ara h 1, Ara h 2, Ara h 3, Ara h 8, Ara h 9, Timothy grass allergen components Phl p 1, Phl p 4, Phl p 5B, Phl p 12. Results are expressed as Relative Risk and 95% confidence intervals. Results are expressed as Relative Risk and 95% confidence intervals. 

## 3. Results and Discussion

### 3.1. Clinical Features and Severity of Acute and Chronic Allergic Diseases

192 patients with peanut allergy and a median age of five years (inter-quartile range 2 to 10 years) were studied (Table 1). The median (interquartile range) interval between the reaction and seeing the patient and collecting blood was 15 (3–38) months. Peanut-containing foods causing the reaction were whole nuts in 56%, biscuit/cake/cereal in 17%, chocolates in 16%, and Chinese/Indian/Italian food in 6%. 32% of patients had a history of reacting to other foods, in 15% tree nuts and in 11% milk, egg, or fish. Median (inter-quartile range) onset of symptoms after eating the food was two (1 to 10) minutes. Time from onset to peak symptoms was 15 (10 to 30) minutes. 

63% patients developed symptoms of upper respiratory, lower respiratory, or altered conscious level. 88% suffered from moderate to severe chronic atopic disease (atopic dermatitis, asthma, and or allergic rhinoconjunctivitis). 40% had two of these conditions, and 20% had all three. 

### 3.2. Clinical and Laboratory Parameters Associated with More Severe Allergic Symptoms

The possible associations between respiratory symptoms/altered consciousness and age, gender, presence or absence of chronic atopic diseases and IgE parameters were investigated (Tables 2 and 3). The extent of exposure to peanut-containing food ingested did not correlate with severity of allergic reaction.

Patients with symptoms of pharyngeal edema were significantly older (median (inter-quartile) age 8 (4 to 16) years) than those with no symptoms (3 (1 to 6) years; *P* < 0.001). They were also 3-4 times more likely to have allergic rhinitis (Table 2). An association with asthma was significant only in patients with severe pharyngeal symptoms such as drooling and dyspnea. Sensitization to peanut sIgE or peanut and Timothy grass pollen allergen components (≥0.35 kU_A_/L) was not associated with clinical symptoms of pharyngeal edema, even after adjusting for the potential confounding effects of clinical and laboratory cofactors using multinominal regression analysis. 

Patients with symptoms of acute bronchospasm were significantly older (6 (3 to 13) years) than those with no bronchospasm (3 (1 to 8); *P* < 0.002). They were also significantly more likely to have asthma requiring regular inhaled corticosteroids (Table 3) but not milder asthma requiring intermittent bronchodilators (data not shown). Patients who developed severe wheeze associated with dyspnea were also significantly more likely to have allergic rhinitis. Patients sensitized to Ara h 9, but not other peanut allergen components, were more likely to have symptoms of bronchospasm than those who were not sensitized (26% versus 9%; *P* = 0.05). 73% of the 33 patients who had Ara h 9 ≥0.35 kU_A_/L (median (range): 1.23 (0.41 to 35.7)   kU_A_/L) had bronchospasm compared with 43% of patients who were not Ara h 9 sensitized (*P* = 0.002). Although Ara h 9 sensitized patients were significantly older (8 (4–16) versus 4 (2–8) years; *P* < 0.001), the association between bronchospasm and Ara h 9 sensitization remained significant even after adjusting for age, as well as other potential confounding factors, such as gender, chronic atopic diseases, and cosensitization with other peanut, or Timothy grass pollen allergen components. 

Compared with children (9%), significantly more adults became dizzy (23%) or lost consciousness (50%) (*P* < 0.001). None of the other clinical (gender, history of atopic disease) or peanut sIgE/allergen component IgE showed a significant correlation (data not shown). 

In this cohort, there was also no association between asthma and IgE sensitization to Ara h 9 or any other peanut component (data not shown). Patients with respiratory symptoms or altered consciousness did not have significantly higher peanut specific or peanut allergen component IgE concentrations than patients who did not have these clinical features. Patients with evidence of IgE sensitized to Ara h 1, 2, and 3 storage proteins were not more likely to develop respiratory symptoms or faintness than those who were not sensitized to any one of these components.

**Figure 1:**
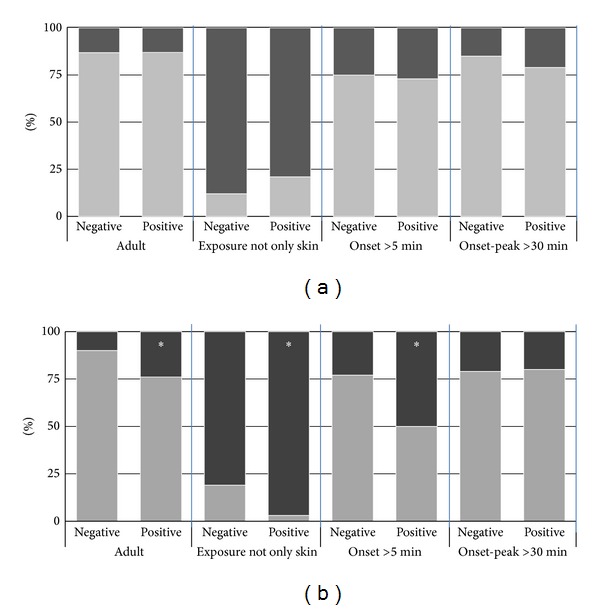
Clinical features of patients who are sensitized to (a) Ara h 2 and (b) Ara h 9. Percentage of patients who: are children (grey bar) or adults (black bar); have a history of skin contact only (grey bar) or ingestion (black bar); onset of symptoms within five minutes of contact with peanut (grey bar) or more than five minutes (black bar); time from onset of symptoms to peak up to 30 minutes (grey bar), over 30 minutes (black bar). “Negative” = Ara h 2 or 9 component negative; “Positive” = Ara h 2 or 9 component positive. *P value < 0.05 using Chi-square test.

Key clinical parameters relating to the allergic reaction were compared in patients sensitized to Ara h 2 and Ara h 9 (Figure 1). Patients who were sensitized to Ara h 2 were not significantly older, more likely to have ingested peanut rather than just have skin contact, have a shorter time to onset or peak reaction than those who were not sensitized (Figure 1(a)). In contrast, significantly more Ara h 9 sensitized patients had a history of ingestion rather than just skin contact with peanut (*P* < 0.05), and 50% of Ara h 9 sensitized patients had an onset of symptoms within five minutes of contact compared with only 23% of patients who were not sensitized (*P* < 0.005). Time from onset to peak symptoms was however not significantly different in Ara h 9 sensitized and nonsensitized patients. 

### 3.3. Cross-Sensitization between Peanut and Pollen Allergen Components

**Figure 2:**
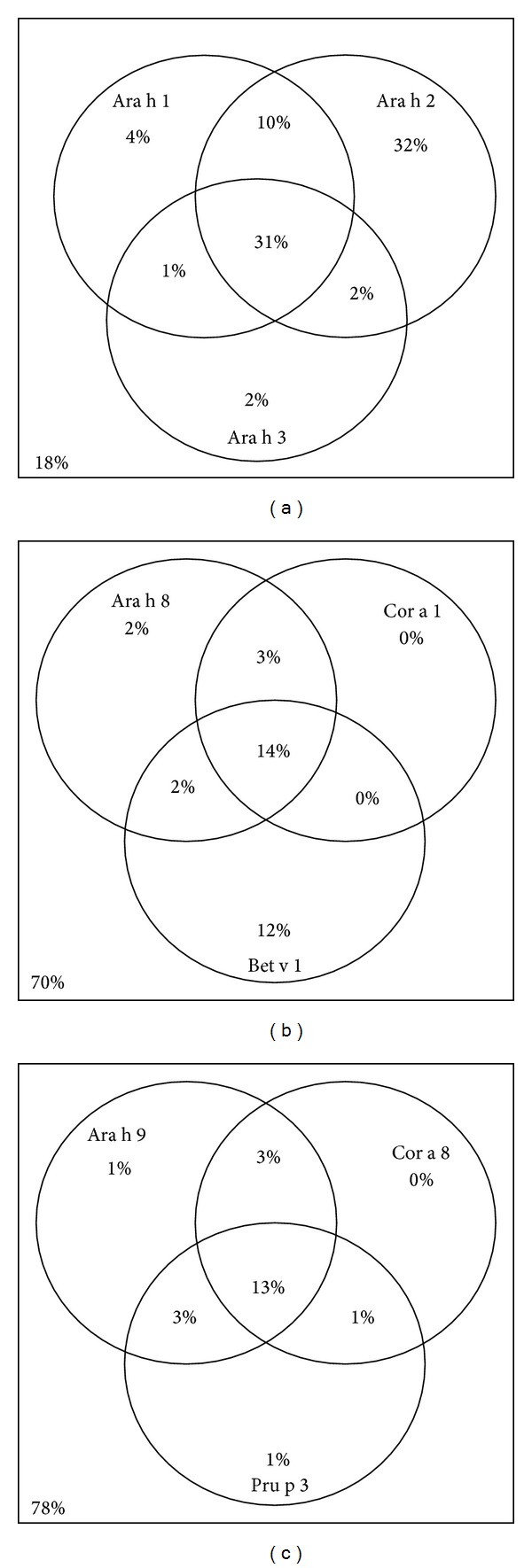
Venn diagrams illustrating the percentage of patients sensitized to IgE allergen components. (a) It shows percentage of patients sensitized to Ara h 1, 2, and 3. (b) It shows the percentage of patients sensitized to the PR-10 proteins Ara h 8, Bet v 1, and Cor a 1. (c) It shows the percentage of patients sensitized to nonspecific lipid transfer proteins Ara h 9, Pru p 3, and Cor a 8.

84% of patients were peanut specific IgE positive and of this group, 82% were Ara 1, 2, or 3 positive. 75% were Ara h 2 positive, 46% were Ara h 1 positive, and 36% were Ara h 3 positive (Figure 2(a)). All of the 33 patients (18%) who showed no IgE sensitization to Ara h 1, 2, and 3 were peanut skin prick test positive with wheals of 4–25 mm. 

Sensitization to Ara h 8 and 9 was less common, being 21% and 20% of the total cohort, respectively. The Ara h 8 component of peanut is a PR-10 pathogenesis-related protein, which may cross-react with other PR-10 proteins, such as Bet v 1 from Birch pollen and Cor a 1 from hazelnut. There was a strong correlation between sensitization to these three components. 14% of the total cohort was positive to all three allergen components, and 70% were negative to all three components, leaving only 16% where there was any discordance (Figure 2(b)). Of the patients that were Ara h 8 sensitized, the median (IQR) concentrations were higher for both Bet v 1 13.4 (2.8–22.7) kU_A_/L and Cor a 1 9.7 (5.3–16.7 kU_A_/L) components than for Ara h 8 (2.3 (1.3–7.0)   kU_A_/L). Although PR-10 pathogenesis-related proteins, 80–100% of patients were not considered sensitized to Ara h 8 were also sensitized to Ph p 1, Phl p 4, Phl p 5B, and Phl p 12 components of Timothy grass pollen (*P* < 0.005) (Table 4).

Ara h 9 is a nsLTP, as are Pru p 3 (peach allergen component) and Cor a 8 (hazelnut allergen component). 13% of the cohort was positive to all three allergen components, and 78% were negative to all three components, leaving only 9% where there was any discordance (Figure 2(c)). Of the patients that were Ara h 9 sensitized, the median (IQR) concentrations were higher for both Pru p 3 5.1 (1.7–10.5)   kU_A_/L and Cor a 8 1.8 (0.5–3.4 kU_A_/L) components than for Ara h 9 (0.8 (0.4–1.8) kU_A_/L). Although an nsLTP protein was not considered, 50% of patients sensitized to Ara h 9 were also sensitized to the Phl p 12 component of grass pollen, while 92% of patients who were not sensitized to Ara h 9 were negative to Phl p 12 (*P* < 0.001) (Table 4). None of the other Timothy grass pollen components showed such a significant association with Ara h 9. 

### 3.4. Interpretation and Relationship of the Results to Previous Studies

In patients with peanut allergy, the nsLTP peanut component Ara h 9 was associated with a significantly higher risk of bronchospasm but not pharyngeal edema or altered consciousness. In this cohort, there was no association between reaction severity and other peanut components (Ara h 1, 2, 3, and 8). In particular, although Ara h 2 was the predominant allergen component, Ara h 2 sensitization did not predict severity of respiratory or circulatory symptoms. 

Previous studies have suggested that Ara h 9 is a more important peanut allergen in Mediterranean countries than other parts of the world [19]. Although only one in ten patients in our cohort was sensitized to Ara h 9, it also appears to be clinically relevant to Northern European populations. The observed association between Ara h 9 and peanut-associated bronchospasm supports the growing appreciation of LTP-associated food-induced anaphylaxis as an important subgroup of food allergy [20–27]. This contrasts with oral allergy syndrome associated with sensitization to the heat and enzyme labile PR-10 allergens such as Bet v 1-like allergen components [28]. The latter group of patients often also have Birch-pollinosis, while the former group may not. In our study, sensitization to Ara h 8 (a Bet v 1 homologue) was not associated with milder symptoms, probably because of the overlap between sensitization to this and other peanut components. As only 6% of the 192 patients in our cohort were sensitized to Ara h 8 alone, subgroup analysis was not feasible. 

Protein allergens within the LTP family are known to have high levels of sequence homology and thus IgE cross-sensitization. An example is Ara h 9 derived from peanut and Pru p 3 from peach where the sequence homology is 62–68% [17]. It is therefore not surprising that we found 91% concordance in cross-sensitization between three LTPs: Ara h 9, Pru p 3, and Cor a 8, the LTP from hazelnut. Only 5 (3%) of patients in this cohort had a clinical history of allergy to hazelnut and only one had reacted to peach, indicating that peanut rather than peach or hazelnut is likely to be triggering the cross-sensitization to these nsLTPs. Similarly high sequence homology is found in PR-10 proteins and we found that the IgE sensitization concordance of the PR-10 homologues of peanut (Ara h 8), hazelnut (Cor a 1), and birch pollen (Bet v 1) was just as high at 86%. Although this study found that the peanut Ara h 9 IgE component concentration was lower than food and pollen homologs, direct comparisons between allergens should be made cautiously, as specific IgE thresholds associated with risk of clinical reactions are known to vary [29].

Timothy grass pollen allergen components are also known to cross-react with peanut components, but in this study Timothy grass pollen components did not lead to any confounding effects in relation to the association between Ara h 9 and bronchospasm. Furthermore, there was no significant association detected between Phl p 12 the Timothy grass pollen component associated with Ara h 9 sensitization and severity of the allergic reaction, presence of concomitant atopic disease, age of the patients or timing of onset of the reaction. 

As Ara h 9 sensitization was negative in 80% of the peanut allergic patients, measurement of this allergen component has a low sensitivity in predicting severe reactions to peanut. Ara h 9, and Ara h 1, 2, and 3 are antigenically stable, even after cooking, and therefore the fact that 39% of peanut was ingested in a cooked or processed form is unlikely to have influenced the results of this study [30]. It has previously been shown that evidence of sensitization to the major peanut allergens remains stable over 20 months [31]. There is no evidence of significant degradation of antigenic components after prolonged freezing, thus storage of the samples is unlikely to be a significant confounding factor. Additional subanalyses, which included only patients having a total IgE of >60 kU/L, gave similar results and thus provided evidence that the lack of correlation was not due to false negative results because of low/normal total IgE concentrations in the cohort. None of the patients were taking medication which might have exaggerated the clinical symptoms and thus confounded the results. 

Although formal oral challenge to peanut is a more objective measure of peanut allergy than clinical history taking, the former procedure is designed to start with amounts of the allergen that are unlikely to trigger a reaction, and it is classically discontinued at the first definite signs of allergy, usually urticaria or local facial angioedema rather than anaphylaxis. Thus, it is not ideal for determining factors associated with severe allergic reactions/anaphylaxis. Clinical history relies on recall of past events, and thus there may be concerns about its accuracy. The proformas used in this study and the fact that the two allergists did a number of clinics together to standardize data collection helped to keep any bias or variation in the way the information was collected from this cohort to a minimum. 

In keeping with our previous study, clinical features of pharyngeal edema were more common particularly in patients with allergic rhinitis and bronchospasm more common in patients with asthma [5]. We would therefore recommend that all patients with peanut allergy have their asthma and allergic rhinitis management optimised as a possible therapeutic measure to reduce the risk of peanut-induced anaphylaxis.

## 4. Conclusions

This study shows that IgE sensitization to the nsLTP Ara h 9 may be an important factor in determining the severity of bronchospasm in some patients with a history of peanut allergy. It is not the only factor as only 26% of patients with symptoms of bronchospasm were Ara h 9 positive and it is not specific as 9% of patients with no bronchospasm were sensitized to this component. In this regard, there is no evidence from this study that component analysis can replace clinical history, or where there is doubt about the history, replace formal oral challenge with peanut. The results do however fit with the growing body of evidence which suggests that LTP components of a number of foods are associated with clinical features of systemic allergic reactions. Our findings need to be verified in additional independent cohorts and if confirmed, the mechanisms linking Ara h 9 to severe allergy should be studied further.

## Figures and Tables

**Table 1:** Clinical characteristics of 192 patients with peanut allergy.

Parameter	Distribution
Demographics	
Age (years)	5 years; 170 (88%) <16 years old
Gender	55% male, 45% female
Extent of exposure	16% skin contact only, 24% taste/lick, 28% up to one teaspoon, 6% over one teaspoon, 26% information unavailable
Acute symptoms (nonanaphylactic)	
Urticaria	nil 42% mild 27% generalized 30%
Peripheral angioedema	nil 25% face 66% generalized 8%
Vomiting	nil 62% once 26% recurrent 12%
More severe allergic reaction (respiratory symptoms or altered consciousness)	
Pharyngeal edema	nil 25% hoarse 16% drooling/dyspnea 25%
Bronchospasm	nil 52% wheezy 24% severe dyspnea 25%
Reduced consciousness	nil 84% dizzy 11% unconscious 5%
Chronic atopic disease	
Atopic dermatitis	72%
Asthma	57%
Allergic rhinitis	37%
History of reactions to other foods	
None	68%
Tree nuts	15%
Other legumes (peas, lentils)	3%
Cow's milk egg, fish	11%
Fruit (apple, peach, grape)	3%

**Table 2:** Clinical and IgE parameters of 192 patients with peanut allergy, with or without symptoms of pharyngeal edema.

	No symptoms	Hoarse voice		Drooling/dyspnea	
Clinical characteristics					
Number	112	32		48	
% adults	4%	22%	6.3 (1.6–25)*	27%	11.9 (3.2–44)**
% male gender	58%	44%	1.3 (0.5–3.5)	54%	0.7 (0.3–1.6)
AD	79%	59%	0.6 (0.2–1.6)	65%	0.4 (0.2–1.0)
Asthma	54%	55%	1.4 (0.5–3.8)	73%	2.9 (1.2–7.0)*
Rhinitis	24%	50%	3.4 (1.4–8.1)*	60%	4.3 (2.0–9.5)**
Peanut allergen specific IgE					
peanut	84%	78%	1.6 (0.4–7.0)	82%	0.6 (0.2–2.7)
Ara h 1	42%	37%	0.6 (0.1–2.4)	35%	1.3 (0.4–4.0)
Ara h 2	65%	57%	0.4 (0.1–1.5)	60%	0.8 (0.2–2.8)
Ara h 3	29%	35%	2.4 (0.8–4.7)	21%	0.7 (0.2–2.2)
Ara h 8	20%	17%	0.7 (0.2–2.5)	19%	0.4 (0.1–1.4)
Ara h 9	17%	12%	0.6 (0.4–1.3)	25%	1.7 (0.5–5.4)
Timothy grass pollen allergen specific IgE					
Phl p 1	53%	60%	1.9 (0.5–8.3)	49%	0.5 (0.2–1.6)
Phl p 4	65%	62%	0.5 (0.1–2.2)	68%	1.2 (0.4–3.8)
Phl p 5B	41%	48%	1.5 (0.4–6.1)	47%	2.2 (0.7–6.8)
Phl p 12	54%	65%	0.5 (0.1–2.8)	39%	0.2 (0.1–1.5)

Data are given as percentage of group. Specific IgE ≥ 0.35 k_A_U/L is defined as “sensitized.” Statistical analysis was performed using multinomial logistic regression analysis quoting Relative Risk (95% confidence interval). *P value < 0.05, **P value < 0.005.

**Table 3:** Clinical and IgE parameters of 192 patients with peanut allergy, with or without symptoms of bronchospasm.

	No symptoms	Wheeze		Wheeze and dyspnea	
Clinical characteristics					
Number	98	46		48	
% adults	6%	15%	3.0 (0.8–11.1)	23%	3.8 (1.2–12.6)*
% male gender	53%	61%	0.5 (0.2–1.1)	52%	0.7 (0.8–1.6)
AD	75%	70%	0.8 (0.3–2.0)	69%	0.7 (0.3–1.7)
Asthma	49%	71%	3.3 (1.3–8.0)*	65%	2.3 (1.0–5.1)*
Rhinitis	28%	39%	1.4 (0.6–3.4)	54%	3.6 (1.5–8.7)**
Peanut allergen specific IgE					
peanut sIgE	83%	78%	0.3 (0.1–1.2)	81%	1.1 (0.3–4.0)
Ara h 1 sIgE	37%	41%	1.1 (0.4–3.4)	41%	1.5 (0.5–4.8)
Ara h 2 sIgE	63%	64%	1.7 (0.5–6.5)	57%	0.4 (0.1–1.4)
Ara h 3 sIgE	24%	27%	0.8 (0.2–2.5)	38%	2.3 (0.8–7.1)
Ara h 8 sIgE	15%	20%	0.8 (0.3–2.5)	25%	0.9 (0.3–2.6)
Ara h 9 sIgE	9%	25%	6.1 (1.9–9.9)**	28%	3.2 (1.1–10.6)*
Timothy grass pollen allergen specific IgE					
Phl p 1	54%	64%	2.3 (0.7–7.4)	39%	0.3 (0.1–1.2)
Phl p 4	64%	69%	1.0 (0.3–3.6)	64%	1.4 (0.4–4.6)
Phl p 5B	45%	44%	0.5 (0.2–5.4)	42%	2.4 (0.4–4.9)
Phl p 12	12%	16%	1.2 (0.3–5.4)	18%	1.1 (0.2–4.9)

Data are given as percentage of group. Specific IgE ≥ 0.35 k_A_U/L is defined as “sensitized.” Statistical analysis was performed using multinomial logistic regression analysis quoting Relative Risk (95% confidence interval). *P value < 0.05, **P value < 0.005.

**Table 4:** Correlation between sensitization to Timothy grass pollen and other allergen components.

Timothy grass pollen component	% of cohort sensitized	Correlation with other allergen components*
Phl p 1	33%	Ara h 8, Cor a 1, Bet v 1
Phl p 4	64%	Ara h 8, Cor a 1, Bet v 1
Phl p 5B	44%	Ara h 8, Cor a 1, Bet v 1
Phl p 7	5%	—
Phl p 12	13%	Ara h 8, Cor a 1, Bet v 1, Ara h 9, Cor a 8, Pru p 3

*P < 0.005 as assessed by Chi-square test.
